# Longitudinal Evidence of Sustained Taurine Deficiency in Inflammatory Bowel Disease

**DOI:** 10.3390/ijms27020725

**Published:** 2026-01-11

**Authors:** Rachele Frascatani, Adelaide Mattogno, Silvia Salvatori, Andrea Iannucci, Irene Marafini, Giovanni Monteleone

**Affiliations:** 1 Department of Systems Medicine, University of Rome “Tor Vergata”, 00133 Rome, Italy; 2 Policlinico Universitario “Tor Vergata”, 00133 Rome, Italy; 3 Department of Biomedicine and Prevention, University of Rome “Tor Vergata”, 00133 Rome, Italy

**Keywords:** Crohn’s disease, ulcerative colitis, IBD, amino acids

## Abstract

Inflammatory Bowel Diseases (IBD), most notably ulcerative colitis (UC) and Crohn’s disease (CD), are long-standing disorders driven by dysregulated immune responses within the gastrointestinal tract and characterized by several metabolic disturbances, which are believed to influence disease progression. We have recently shown that a systemic deficiency of taurine, a semi-essential amino acid with anti-inflammatory properties, marks IBD. To characterize the temporal dynamics and determinants of taurine deficiency in IBD, we conducted a prospective longitudinal study assessing serum taurine levels in a cohort of 47 patients with IBD compared with 33 healthy controls. Serum taurine concentrations were measured at baseline and after a median follow-up period of 45 months using ELISA. Patients were stratified by disease subtype (UC and CD), age group, and clinical activity status at baseline and follow-up. Serum taurine levels were significantly lower in IBD patients at both baseline and the end of follow-up (*p* < 0.05), and remained stable over time within the CD and UC cohorts. In healthy individuals, but not in IBD patients, taurine concentrations declined with age, suggesting that age-related metabolic regulation of taurine is altered in the context of chronic intestinal inflammation. Stratification by disease activity revealed that taurine deficiency was present in both active and inactive IBD, particularly among younger patients, and differences between active and inactive disease were minimal. These findings indicate that the persistent reduction in serum taurine in IBD is independent of age, disease subtype, or clinical activity, and remains relatively constant over time across most patient subgroups, suggesting an underlying alteration in taurine metabolism or homeostasis associated with IBD pathophysiology. Further investigation is needed to elucidate the mechanisms linking taurine dysregulation to IBD progression.

## 1. Introduction

Inflammatory Bowel Diseases (IBD), primarily ulcerative colitis (UC) and Crohn’s disease (CD), are long-standing disorders driven by dysregulated innate and T- and B-cell-mediated adaptive immune responses within the gastrointestinal tract [1,2,3,4,5]. This chronic inflammatory state constitutes significant challenges for treatment, long-term disease management, and patients’ quality of life. Although IBD clinical presentation and underlying mechanisms vary, both CD and UC share a complex interplay between genetic susceptibility, environmental triggers, and microbial factors that contribute to chronic intestinal inflammation [6,7,8,9,10]. Increasing attention has been directed toward the metabolic disturbances that accompany IBD and may influence disease progression [11,12,13].

One such metabolic alteration involves taurine, a sulfur-containing amino acid known for its anti-inflammatory, antioxidant, and cytoprotective roles [14,15]. Thanks to its properties, taurine is thought to play an important role in modulating gut inflammation and maintaining epithelial barrier function, both of which are compromised in IBD [16,17,18]. Taurine is primarily synthesized in the liver from cysteine and methionine via the cysteine sulfinic acid pathway, and can also be obtained from dietary sources such as meat and fish. It participates in bile acid conjugation, osmoregulation, and the modulation of calcium signaling, and is rapidly taken up by tissues through specific transporters, highlighting the tight regulation of its metabolism [19]. Reduced circulating taurine has been reported in individuals with IBD, yet the factors driving this depletion and its temporal characteristics remain poorly understood. Earlier work from our group suggested that taurine levels may fluctuate with disease activity in UC but not in CD, suggesting potential differences in metabolic responses between the two disease subtypes [14].

To clarify the evolution and determinants of taurine deficiency in IBD, this longitudinal study tracks serum taurine concentrations in patients with UC and CD and compares them with those in healthy individuals. By examining changes over time and across age groups, we aim to determine whether taurine depletion reflects active inflammation or indicates a persistent metabolic alteration in IBD. Insights from this work may deepen our understanding of IBD pathophysiology and help identify novel metabolic targets for future therapeutic strategies, including potential metabolic interventions or taurine-based supplementation approaches.

## 2. Results

### 2.1. Patients’ Characteristics

The study population included 18 UC patients, 29 CD patients, and 33 healthy control participants (Table 1). At baseline, the median age was 49.5 years (range: 21–64 years) among patients with UC and 41 years (range: 18–76 years) among those with CD. Eleven UC patients (61%) and 11 CD patients (37.9%) were female. Overall, 21 (72%) CD patients and 10 (55%) UC patients were younger than 50 years. Age was included in subsequent analyses to assess its potential association with serum taurine concentrations. In the CD group, 6 patients (21%) had colonic involvement, 12 (41%) had disease limited to the distal ileum, and 11 (38%) had both ileal and colonic involvement. In the UC group, 10 (55.5%) patients had left-sided colitis (E2), and 8 (44.5%) had extensive colitis (E3).

At baseline, 9 (31%) CD patients and 9 (50%) UC patients received mesalamine, while 2 (11%) UC patients and 2 (7%) CD patients were treated with steroids. Biologic agents were used by 14 (48%) CD patients and 6 (33%) UC patients, whereas immunosuppressive therapy was used by 3 (10%) CD patients. Additionally, one CD patient was receiving antibiotic therapy, and one UC patient was not receiving any treatment. At the end of follow-up, 6 (21%) CD patients and 12 (67%) UC patients were receiving mesalamine, and steroid therapy was documented in only one (3.5%) CD patient. Biologic therapy was used by 21 (72.5%) of the CD patients and 6 (33%) of the UC patients. One CD patient was receiving antibiotics.

In the control group, the median age was 37 years (range: 23–72 years), and 22 participants (67%) were female. Among patients with IBD, 25 individuals (53%) were women. The median age of the IBD cohort was 44 years (range: 18–76 years) at baseline and 47 years (range: 22–80 years) at the end of follow-up, with no significant differences compared with controls (*p* = 0.768 and *p* = 0.146, respectively).

The UC and CD groups did not differ significantly in terms of age or sex distribution. At baseline, 13 CD patients (44.8%) and 13 UC patients (72%) had clinically active disease. Among these patients, 35 (23 CD and 12 UC) maintained the same disease activity, 10 (4 CD and 6 UC) patients changed from active to inactive, and 2 CD patients changed from inactive to active.

Regarding therapy, 29 patients (17 CD and 12 UC) were on the same treatment at baseline and follow-up. Six patients (5 CD and 1 UC) switched from mesalamine-based therapy to biologic therapy, and three patients (1 CD and 2 UC) switched in the opposite direction. Three CD patients who were receiving immunosuppressants and one CD patient who was receiving steroids were on biologic therapy at follow-up. In addition, the UC patient who was not receiving any therapy at baseline was receiving biologic therapy at the time of the second blood draw. Finally, two UC patients who were receiving steroids and one CD patient who was receiving antibiotics switched to mesalamine-based therapy, and one CD patient transitioned from mesalamine therapy to antibiotic treatment. Given the very small number of cases and the absence of formal statistical testing, no inference was made regarding potential changes in taurine levels associated with these therapeutic shifts.

### 2.2. Serum Taurine Levels Are Persistently Reduced in IBD

**Figure 1:**
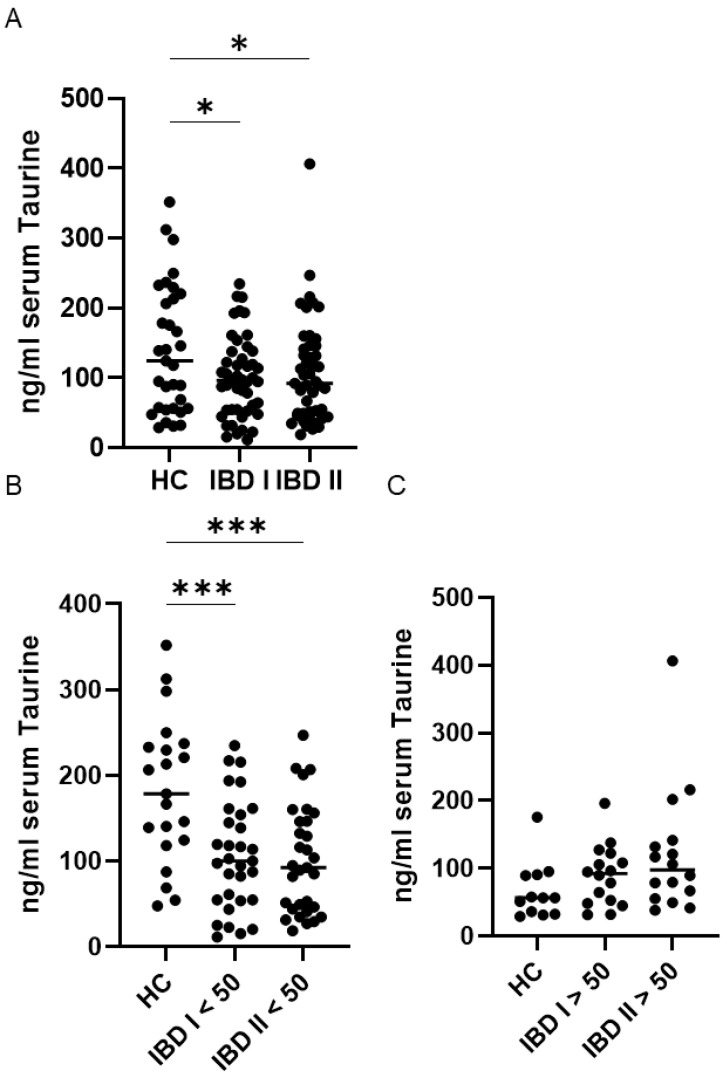
Sustained reduction in serum Taurine levels in inflammatory bowel disease (IBD) patients younger than 50 compared with healthy controls (HC). (**A**) Serum taurine concentrations were measured in 33 HC and 47 patients with IBD at baseline (I) and after a median follow-up of 45 months (II). Each data point represents an individual participant, and horizontal lines denote the median value for each group. * *p* < 0.05. IBD patients and HC were divided into two subgroups based on age. Panel (**B**) includes individuals younger than 50 years, whereas panel (**C**) shows those aged 50 years or older. Each data point represents the serum taurine concentration of an individual participant, and horizontal bars indicate the median for each group. *** *p* < 0.001.

Serum taurine levels were markedly lower in patients with IBD at both the baseline and the end-of-follow-up evaluations compared with healthy controls. Within the IBD cohort, taurine levels remained stable over time, with no significant differences observed between the initial and final measurements (Figure 1A), as assessed by a paired *t*-test. Given that circulating taurine levels decline with age in humans [20], we compared serum taurine concentrations in individuals younger than 50 years with those aged 50 years or older. Among healthy controls, as expected, median taurine levels were significantly higher in individuals under 50 compared to those over 50 (*p* < 0.05). In contrast, the IBD cohort showed no age-related differences, with similar serum taurine concentrations observed in younger and older patients at both baseline and at the end of follow-up (Figure 1B,C).

Furthermore, in IBD patients, serum taurine levels remained stable over time, both in individuals younger than 50 years and in those aged 50 years or older (Figure 1B,C).

**Figure 2:**
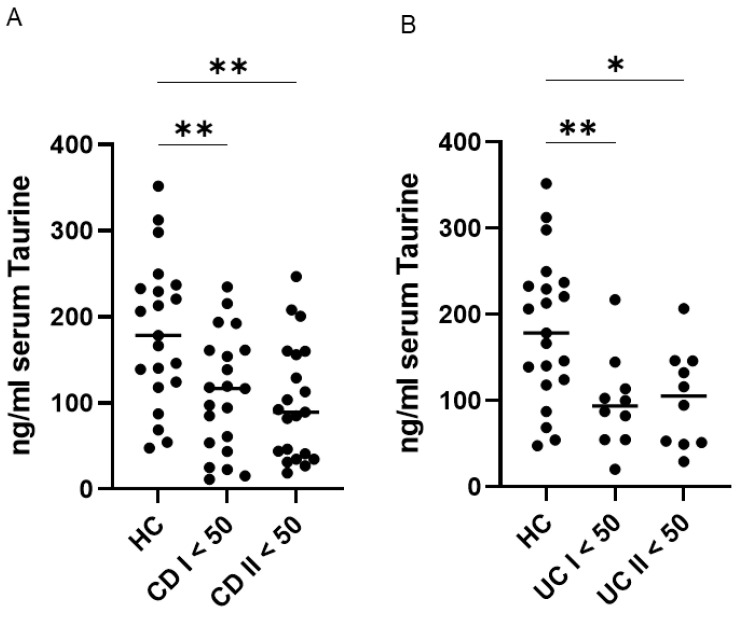
Persistent reduction in serum taurine levels among patients with ulcerative colitis (UC) and Crohn’s disease (CD) younger than 50 years compared with healthy controls (HC). (**A**,**B**) Serum taurine concentrations were assessed in 21 HC, 21 patients with CD, and 10 patients with UC younger than 50 years at baseline and after a median follow-up of 45 months. Each data point represents an individual participant, and horizontal bars indicate the median for each group. * *p* < 0.05; ** *p* < 0.01.

Further analyses demonstrated that patients with CD and UC exhibited significantly lower serum taurine levels compared with healthy controls at both baseline and the end of follow-up. No significant changes in taurine concentrations were observed over time within either disease group (Figure 2).

### 2.3. Persistent Reduction in Serum Taurine Is Independent of Disease Activity

In our previous work, we observed that lower serum taurine levels in IBD patients were not significantly affected by body mass index, IBD subtype, or the intake of taurine-rich foods (such as seafood and dark-meat poultry) or taurine-containing energy drinks [14]. Although serum taurine concentrations were lower in IBD patients with clinically active disease compared to those in remission, this difference was evident only in UC, not in CD, suggesting that factors beyond disease activity may contribute to taurine deficiency in IBD [14].

**Figure 3:**
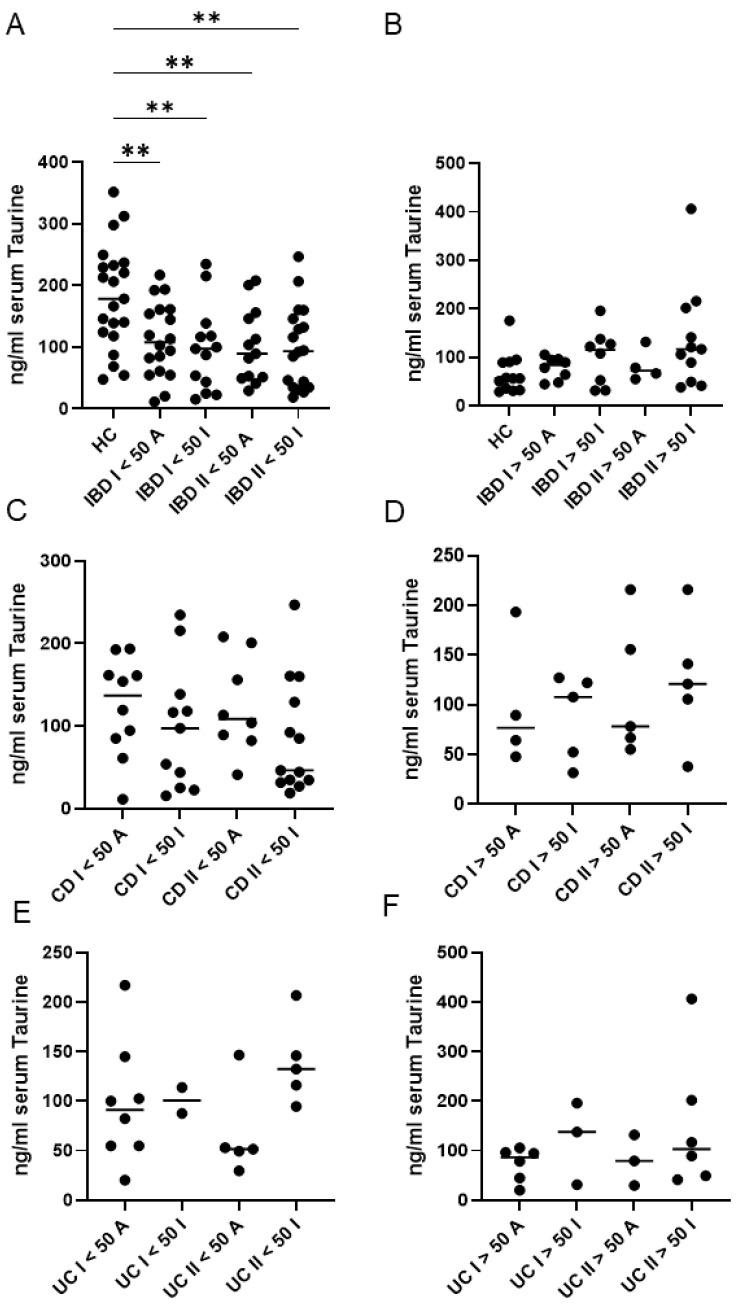
Serum taurine levels in healthy controls (HC), and both ulcerative patients (UC) and Crohn’s disease (CD) patients, according to their age (younger or older than 50 years) and disease activity. (**A**,**B**) Taurine was measured in serum samples of 33 HC and 47 IBD patients at baseline (I) and after a median follow-up period of 45 months (II). HC and IBD patients were stratified into subgroups based on their age (younger or older than 50 years) and disease activity [active (A) or inactive (I)]. ** *p* < 0.01. (**C**,**D**) Taurine was measured in serum samples of 29 CD patients at baseline (I) and after a median follow-up period of 45 months (II). CD patients were stratified into subgroups based on their age (younger or older than 50 years) and disease activity [active (A) or inactive (I)]. (**E**,**F**) Taurine was measured in serum samples of 18 UC patients at baseline (I) and after a median follow-up period of 45 months (II). UC patients were stratified into subgroups based on their age (younger or older than 50 years) and disease activity [active (A) or inactive (I)]. Each data point in the graph represents the serum taurine concentration of an individual participant, and the horizontal bars indicate the median value for each subgroup.

When IBD patients were stratified according to disease activity, both active and inactive patients exhibited significantly lower taurine levels than healthy controls, particularly among those younger than 50 years (Figure 3A,B). In contrast, no significant differences in taurine concentrations were detected between active and inactive patients at baseline or at the end of follow-up, in either age group (<50 years or ≥50 years) (Figure 3A,B).

Furthermore, a more detailed assessment involved stratifying patients not only by clinical activity and age but also by IBD type (Figure 3C–F). This analysis confirmed the absence of correlation between activity and taurine levels for both conditions at baseline or at the end of follow-up, although a trend seemed to be present in UC (Figure 3E,F).

## 3. Discussion

This study provides evidence that serum taurine concentrations are consistently lower in patients with IBD compared with healthy controls. Importantly, this deficiency was observed at both baseline and follow-up assessments, suggesting that taurine depletion represents a persistent characteristic of IBD rather than a transient response to acute disease activity.

In healthy individuals, serum taurine decreased with age, consistent with previous reports linking reduced taurine to aging and age-associated diseases [20,21,22]. In contrast, age-related differences were not observed in the IBD cohort: younger patients (<50 years) already exhibited significantly lower taurine levels compared with age-matched controls, and older patients (≥50 years) did not show further decline. These results indicate that taurine deficiency in IBD is independent of age and cannot be attributed to the slightly younger mean age of the control cohort.

Within the IBD population, both CD and UC patients had reduced levels compared with controls, and taurine did not differ significantly between baseline and follow-up in either group. Stratification by clinical disease activity revealed that active IBD patients had similar levels of taurine to those measured in patients who were in remission. Moreover, within each age group, active and inactive patients showed comparable taurine concentrations. Collectively, these results suggest that taurine deficiency in IBD is not driven by clinical disease activity. Potential factors contributing to taurine reduction were explored in our previous study, including body mass index, dietary intake of taurine-rich foods, and consumption of taurine-containing energy drinks [14]. None of these factors significantly influenced circulating taurine levels. It is therefore plausible that the taurine deficiency may stem from chronic metabolic adaptations to long-standing intestinal inflammation, increased utilization in oxidative-stress defense pathways, altered microbial metabolism, and/or impaired endogenous biosynthesis. Future investigations evaluating mucosal taurine metabolism, as well as the activity of enzymes such as cysteine sulfinic acid decarboxylase, are warranted to clarify whether defects in this biosynthetic route directly contribute to systemic taurine reduction in IBD.

Metabolites function as key regulators of immune activity, intestinal homeostasis, and inflammatory signaling, thereby linking metabolic disturbances to IBD pathology [23,24,25,26,27]. Among these metabolites, amino acids play particularly complex and influential roles in shaping immune responses and maintaining gut function [28,29,30,31]. Tryptophan, an essential amino acid, is of special interest because it can be processed through three major metabolic routes, the kynurenine, serotonin, and microbial indole pathways, each yielding bioactive compounds with distinct immunological effects [32,33]. In the kynurenine pathway, metabolites can activate the aryl hydrocarbon receptor (AhR), fostering regulatory T-cell development and limiting the proinflammatory actions of pathogenic T cells, ultimately helping to restrain intestinal inflammation [34]. Yet, individuals with IBD frequently show disruptions in tryptophan metabolism, including increased kynurenine levels that correlate with heightened disease activity and inflammatory burden [35,36,37]. Microbial conversion of tryptophan also generates indole derivatives with protective functions. For example, indole-3-propionic acid (IPA) has been shown to strengthen epithelial barrier integrity and reduce inflammation via AhR-dependent mechanisms [38,39]. The clinical significance of taurine reduction in IBD is supported by its known roles in modulating inflammation, protecting against oxidative stress, regulating mitochondrial function, and maintaining epithelial barrier integrity, which are central to IBD pathophysiology [40,41]. Indeed, previous experimental studies have demonstrated that taurine supplementation can mitigate gut inflammation and restore immune balance [18,42]. It is therefore conceivable that persistent taurine deficiency may contribute to disease progression through increased oxidative stress, endothelial dysfunction, and immune dysregulation. These results align with earlier reports documenting decreased circulating taurine concentrations in individuals with Parkinson’s disease [43]. Moreover, in type 2 diabetes mellitus, several studies report significantly reduced serum taurine levels, which have been associated with complications such as diabetic neuropathy and endothelial dysfunction [44,45,46]. Chronic kidney disease and patients on dialysis frequently show reduced circulating taurine, likely due to increased renal loss and altered metabolism [47,48,49]. Experimental and clinical data suggest that taurine depletion in these contexts may contribute to oxidative stress and inflammatory dysregulation, while supplementation could exert cytoprotective effects [40,41,50,51]. In contrast, some studies of acute coronary syndromes indicate elevated serum taurine, possibly reflecting tissue release or compensatory protective mechanisms [52,53]. In this context, it is also noteworthy that a recent study reported a marked increase in taurine and hypotaurine metabolic pathways in the intestinal tissues of patients with IBD compared with healthy individuals. The researchers also observed that higher intestinal taurine levels were associated with reduced disease severity. Supporting this protective role, deletion of the taurine-biosynthesis gene CSAD worsened dextran sodium sulfate-induced colitis in mice, whereas taurine supplementation mitigated the intestinal inflammation [54], further reinforcing the potential relevance of taurine metabolism in IBD pathophysiology.

## 4. Materials and Methods

### 4.1. Study Population and Data Collection

This prospective observational study was conducted at a single tertiary referral center specializing in inflammatory bowel disease (IBD) care (Tor Vergata University Hospital, Rome, Italy). Adult outpatients with IBD attending scheduled clinic visits were consecutively recruited, and blood samples were collected immediately after each consultation at baseline and at study completion, consistently in the morning to ensure uniformity of sampling. IBD serum samples were collected at baseline and after a median follow-up period of 45 months, while healthy control samples were obtained at a single time point. The study population was derived from our previously investigated cohort [14]; the longitudinal sample included only patients who returned for follow-up and had paired serum samples available, while those no longer eligible for reassessment were excluded. For each participant, data and clinical information were collected on IBD subtype, Montreal classification, clinical status at assessment, and current treatments. Disease extent was assessed using endoscopy and, for CD patients, small bowel ultrasound and/or magnetic resonance enterography performed before or at the time of serum sampling.

Clinical activity was evaluated using the Partial Mayo Score (PMS) for UC and the Harvey–Bradshaw Index (HBI) for CD. The same team of IBD specialists conducted all patient assessments at both baseline and follow-up visits, ensuring methodological consistency over time. Active disease was defined as PMS ≥ 2 for UC or HBI ≥ 5 for CD. Serum samples were collected from healthy controls with no personal or family history suggestive of IBD at a single time point. Written informed consent for the scientific, anonymized use of clinical data was obtained from all participants at enrollment. Individuals under 18 years of age or those unable to provide informed consent (e.g., because of language barriers) were excluded. The study received approval from the local ethics committee (protocol N.29091/2022).

### 4.2. Enzyme-Linked Immunosorbent Assay

Serum taurine concentrations were determined using a commercially available competitive ELISA kit (Abbexa, Cambridge, UK). For each assay, 50 μL of serum were measured for absorbance at 450 nm with a DTX 880 multimode reader (Beckman Coulter, Milan, Italy). The kit has the following technical specifications: test range, 12.35–1000 ng/mL; sensitivity, <4.39 ng/mL. According to the manufacturer’s validation data, the assay is suitable for use across multiple biological matrices, including serum, plasma, and other biological fluids. Quantification was performed by comparing sample absorbance values with a standard calibration curve generated from serially diluted reference standards, and all samples were consistently analyzed in duplicate to ensure measurement reliability.

### 4.3. Statistical Analysis

Categorical variables were reported as percentages, and continuous variables were summarized as medians and ranges. Comparisons between independent groups were performed using unpaired *t*-tests, and overall differences among multiple groups were assessed using one-way ANOVA. These methods were consistently applied to comparisons between controls and IBD subgroups (UC and CD), or among the different IBD groups. The only exception concerned paired observations from the same individuals who differed only in sample collection timing (I vs. II), which were analyzed using a paired *t*-test. Statistical analyses were performed using GraphPad Prism, version 9.

## 5. Conclusions

Our study demonstrates that the persistent reduction in serum taurine in IBD is independent of age, disease subtype, or clinical activity, confirming and extending previous observations of taurine deficiency in IBD [14]. This study has several limitations, including the relatively small sample size and the lack of endoscopic or histological data, which limited the evaluation of disease activity to clinical scores. Additionally, disease duration was not considered in the analyses. The observational design also precluded establishing a causal link between taurine deficiency and dietary intake or other lifestyle factors, although we have previously shown no association between serum taurine reduction and such factors [14]. Future studies are needed to investigate the mechanisms of taurine metabolism in IBD, as well as whether taurine supplementation can modulate mucosal inflammation and/or improve intestinal barrier function. Understanding how taurine deficiency contributes to disease progression and pathophysiology may inform new strategies for metabolic intervention and support the development of targeted therapies in IBD.

## Figures and Tables

**Table 1:** Demographic and clinical characteristics of ulcerative colitis (UC) patients and Crohn’s disease (CD) patients at the baseline (I) and the end-of-follow-up evaluations (II).

Characteristics	UC Patients (N = 18)	CD Patients (N = 29)	*p*-Value
Age (years), median [range]	(I) 49.5 [21–64] (II) 52.5 [24–68]	(I) 41 [18–76] (II) 44 [22–80]	(I) 0.196 (II) 0.196
Female gender, n (%)	11 (61)	11 (37.9)	0.212
IBD patients with clinically active disease, n (%)	(I) 13 (72) (II) 7 (39)	(I) 13 (44.8) (II) 11 (37.9)	(I) 0.125 (II) 0.800
Disease localization for UC, n (%): E1 E2 E3	0 (0) 10 (55.5) 8 (44.5)	-	-
Disease localization for CD, n (%): L1 L2 L3	-	12 (41) 6 (21) 11 (38)	-
Disease phenotype for CD, n (%): B1 B2 B3	-	15 (52) 10 (34.5) 4 (13.5)	-

## Data Availability

The original contributions presented in this study are included in the article. Further inquiries can be directed to the corresponding author.
